# Unilateral Arteritic Anterior Ischaemic Optic Neuropathy (AAION) as a Presenting Manifestation of Fatal Giant Cell Arteritis (GCA): A Case Report

**DOI:** 10.7759/cureus.79148

**Published:** 2025-02-17

**Authors:** Nasyitah Yakub, Rosiah Muda, Maizan Yaakub, Julieana Muhammed

**Affiliations:** 1 Department of Ophthalmology and Visual Science, Universiti Sains Malaysia School of Medical Sciences, Kubang Kerian, MYS; 2 Department of Ophthalmology, Hospital Sultanah Nur Zahirah, Kuala Terengganu, MYS; 3 Department of Ophthalmology, Universiti Sains Malaysia, Kubang Kerian, MYS

**Keywords:** giant cell arteritis, ischaemic optic neuropathy, prognosis, temporal artery biopsy, vasculitis

## Abstract

Giant cell arteritis (GCA), also known as temporal arteritis, is a form of vasculitis that primarily affects the large and medium-sized arteries, which can lead to narrowing or blockage of the arteries. When GCA involves vital vessels such as the coronary arteries, aorta, or cerebral arteries, it can pose a serious risk to life. We present a case of a 61-year-old Malay woman with hypertension and chronic kidney disease who presented with a two-day history of sudden-onset visual loss in the right eye preceded by an inferior visual field defect for four days associated with both lower limb pain. Otherwise, there was no fever, headache, scalp pain, or jaw claudication. Her vision was hand movement and 6/6 in the right eye and left eye, respectively, with a positive relative afferent pupillary defect. Fundus examination showed a swollen 'chalky white' pallid appearance of the optic disc with splinter haemorrhage. The examination of the left eye was normal. Both temporal arteries were dilated, tortuous, and non-pulsatile. There were multiple painful necrotic skin lesions over both lower limbs. Blood tests showed a markedly high erythrocyte sedimentation rate (120 mm/hour) and C-reactive protein (144.2 mg/L). The temporal artery biopsy was suggestive of GCA, and the findings were supported by a biopsy from the necrotic skin lesions. She was treated with intravenous methylprednisolone for three days, and her vision maintained hand movement with no involvement of the left eye. She was discharged with oral prednisolone 1 mg/kg; however, one month later, she succumbed to death from cardiac complications.

## Introduction

Giant cell arteritis (GCA), also known as temporal arteritis, is a primary systemic autoimmune disease characterized by the infiltration of moderate-sized and large blood vessels [1]. It predominantly affects older adults, with most patients being over the age of 60. The mean age of onset is around 70 years. Studies indicate that women are more frequently affected than men, with GCA occurring approximately two to three times more often in women.

The incidence of GCA varies by geographic location and population but can be as high as 27 cases per 100,000 people aged over 50 years. The exact cause of GCA remains unknown, but genetic, environmental, and immune system factors are believed to play a role [1].

GCA is considered a medical emergency due to its potential to result in irreversible vision loss in a significant number of cases, affecting over 50% of those who do not receive timely treatment. Vision loss in GCA occurs due to inflammation of the arteries supplying blood to the optic nerve that leads to ischaemic optic neuropathy, resulting in sudden and permanent blindness in one or both eyes [1,2].

Because of the risk of rapid and irreversible vision loss, prompt recognition and treatment of GCA are critical. If GCA is suspected, high-dose corticosteroid therapy is usually started immediately to reduce inflammation and prevent further vascular damage, even before the diagnosis is confirmed by temporal artery biopsy (TAB) or imaging studies. Early intervention can help minimize the risk of severe complications, including vision loss and other potentially life-threatening vascular events [2].

Arteritic anterior ischaemic optic neuropathy (AAION) is the most serious ophthalmological complication of GCA [3] and a potentially fatal complication of GCA that requires rapid treatment [4]. A fatal case of giant-cell arteritis will be reported here in which the patient's eyes were examined clinically and histologically.

This article was previously presented as a meeting abstract at the 38th Asia-Pacific Academy of Ophthalmology Congress on February 23-26, 2023.

## Case presentation

**Figure 1:**
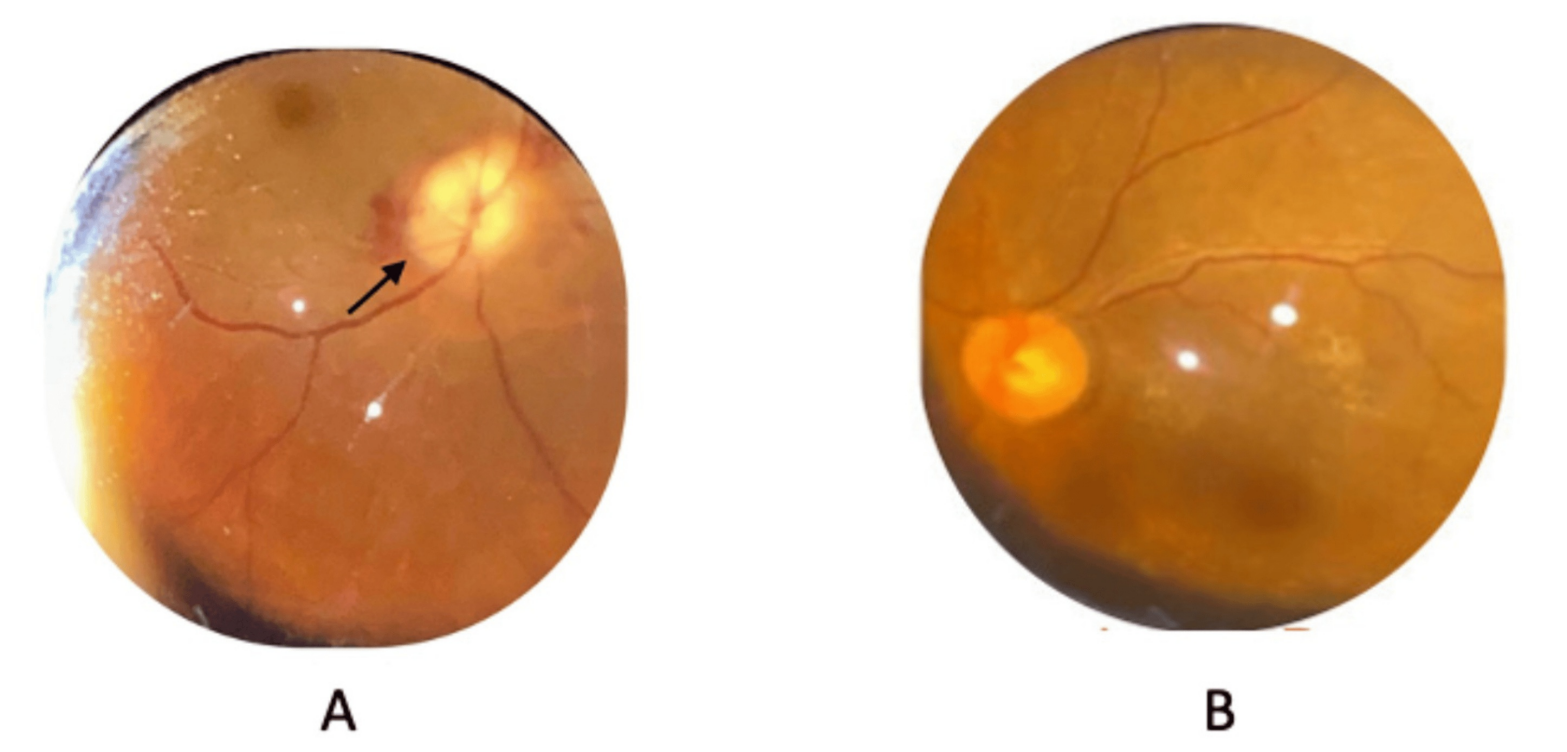
(A) The fundus of the right eye showed a 'chalky white' swelling of the optic disc; (B) normal optic disc of the left eye

A 61-year-old Malay woman with underlying hypertension and chronic kidney disease presented in May 2023 with a two-day history of sudden-onset and painless visual loss in the right eye preceded by inferior visual field defect for four days associated with both lower limbs having severe pain, causing her to be unable to ambulate well even while in admission. Otherwise, there was no fever, headache, chest pain, scalp pain, or jaw claudication. Her vision upon presentation was hand movement and 6/6 in the right eye and left eye, respectively, with a positive relative afferent pupillary defect over the right eye. Both anterior segments were unremarkable. Fundus examination of the right eye showed a swollen 'chalky white' pallid appearance of the optic disc with splinter haemorrhage (Figure 1). The examination of the left eye was normal.

**Figure 2:**
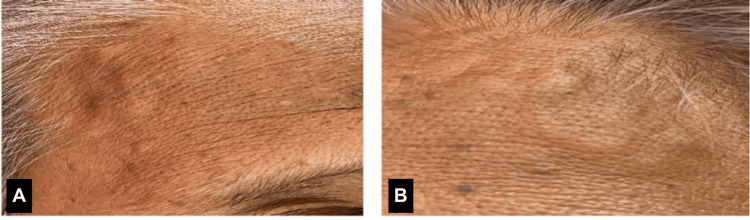
Image showing bilateral temporal arteries were dilated, tortuous, and non-pulsatile (A,B)

**Figure 3:**
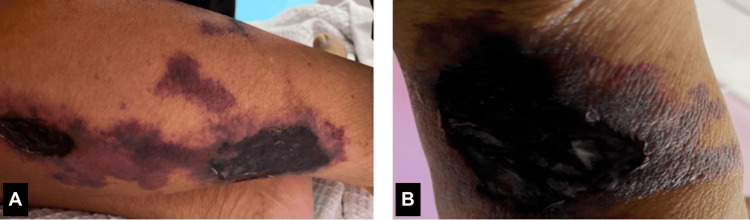
Multiple patches of necrotic skin lesions over the lower limbs (A,B)

The systemic examination revealed that both temporal arteries were dilated, tortuous, and non-pulsatile (Figure 2). There were multiple painful necrotic skin lesions over both lower limbs (Figure 3) caused by the ischaemic cutaneous manifestation of GCA. An urgent referral was made to the dermatology team, and a biopsy was performed. The skin lesions remained stable with no progression.

The laboratory blood tests showed that the erythrocyte sedimentation rate (120 mm/hour) and C-reactive protein (144.2 mg/L) were markedly high, as shown in Table 1.

**Table 1:** Laboratory test results of C-reactive protein (CRP) and erythrocyte sedimentation rate (ESR)

Parameter	Result	Normal Value
C-reactive protein (CRP)	144.2 mg/L	3-10 mg/L
Erythrocyte sedimentation rate (ESR)	120 mm/hour	Men: 0–22 mm/hour; women: 0–29 mm/hour

**Figure 4:**
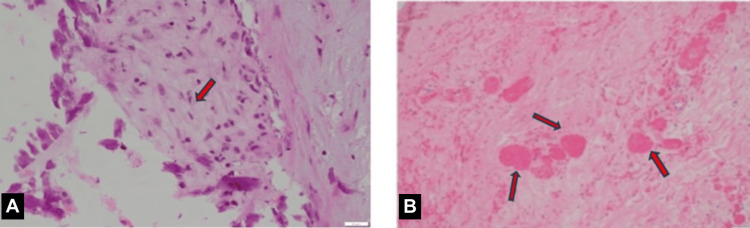
Temporal artery biopsy: (A) the vessel wall is moderately infiltrated by neutrophils and lymphocytes (arrow); (B) numerous intravascular thrombi (arrows) are present within the upper dermis with adjacent vasculitic changes

The case was then co-managed with the vascular team for a TAB that was suggestive of GCA (Figure 4), and the findings were supported by a biopsy from the necrotic skin lesions.

Without further delay, she was treated with intravenous methylprednisolone for three days, and her vision maintained at hand movement with no involvement of the left eye. She was discharged with oral prednisolone 1 mg/kg; however, within one month of treatment, she succumbed to death from cardiac complications.

## Discussion

GCA, also known as temporal arteritis, is a rare form of vasculitis that predominantly affects elderly women. It is most prevalent among Caucasians, particularly those of Northern European descent, and is relatively rare in Asian populations [4]. Our patient presented at the age of 61 years.

The diagnosis of GCA in this case was made based on the American College of Rheumatology (ACR) criteria for GCA, with the patient meeting four out of the five established criteria. The ACR criteria for diagnosing GCA are widely used in clinical practice to help identify the condition, particularly in patients over 50 years of age who present with typical symptoms. The ACR criteria for GCA include the age of more than 50 years old, headache, abnormal temporal arteries, positive TAB, and ESR ≥ 50 mm/first hour [5]. When three or more criteria are met, the test demonstrates a diagnostic sensitivity of 93.5% and a specificity of 91.2% [6].

Cutaneous involvement in our patient was uncommon in GCA, and it is presumed due to ischaemic sequelae such as purpura, necrotic ulcers, or gangrene [7].

Nearly two-thirds of patients with GCA experience ocular symptoms, and up to 30% of these individuals suffer from permanent visual loss. In GCA, the branches of the posterior ciliary arteries are primarily affected. The most common cause of vision loss in GCA is AAION, resulting from vasculitic involvement of the short posterior ciliary arteries [8]. Visual loss in AAION is often profound.

TABs are regarded as the gold standard for diagnosing GCA. Across multiple studies, the pooled sensitivity and specificity of TAB in patients suspected of having GCA were 73% and 94%, respectively, compared to a clinical diagnosis of GCA. The likelihood of obtaining a positive biopsy result is highly influenced by the pretest probability, and some patients with a clinical diagnosis of GCA continue on glucocorticoid therapy even if their biopsy results are negative [9]. Although TAB is a surgical procedure, it is relatively safe, and most patients do not experience any major complications.

Corticosteroids remain the mainstay of treatment and should be initiated immediately to prevent the involvement of the fellow eye [10]. The European League Against Rheumatism (EULAR) guidelines advise initiating remission with a one-month course of high-dose glucocorticoid therapy, using prednisolone at 1 mg/kg per day (up to a maximum of 60 mg/day). For patients presenting with early visual symptoms, the guidelines also recommend intravenous pulsed methylprednisolone, although the exact dosage is not specified. Induction treatment with high-dose pulsed intravenous (IV) methylprednisolone (15 mg/kg for three consecutive days followed by an oral prednisone dose of 40 mg/day) has been suggested for all patients with GCA [11].

Other immunosuppressive treatments have been used at the onset of the disease, mainly to facilitate a quicker tapering of steroids or to help manage severe disease manifestations. However, the outcomes have been inconsistent and generally unsatisfactory [12].

Studies have reported that GCA patients with vision loss have a reduced survival rate when compared with GCA patients with unchanged vision [13], giving the possibility of cerebral arteritis, coronary arteritis, and thromboembolic events [14].

Cerebral arteritis involves inflammation of intracranial arteries, which can result in cerebral ischaemia and increase the likelihood of transient ischaemic attacks (TIAs) or ischaemic strokes. Coronary arteritis, on the other hand, can impair myocardial blood flow, predisposing patients to angina, myocardial infarction (MI), or heart failure. Additionally, GCA can promote endothelial dysfunction, hypercoagulability, and arterial thrombosis, heightening the risk of thromboembolic events [14].

Due to these potentially life-threatening complications, patients with suspected or confirmed GCA require comprehensive vascular assessments and close monitoring. Effective management necessitates a multidisciplinary approach involving rheumatologists, neurologists, cardiologists, and vascular specialists. This collaborative strategy ensures the timely identification of high-risk patients and the implementation of preventive measures, such as aggressive immunosuppressive therapy and cardiovascular risk mitigation.

## Conclusions

GCA is a systemic vasculitis that primarily affects medium- to large-sized arteries and should be strongly suspected in patients over the age of 50 who present with AAION, particularly in the presence of constitutional symptoms, jaw claudication, or scalp tenderness. Prompt recognition of this condition is crucial, as delayed diagnosis and treatment can lead to devastating complications, including irreversible vision loss, cerebrovascular events, and other ischaemic manifestations.

Cutaneous involvement in GCA is uncommon, with necrotic skin lesions representing a rare but serious manifestation of severe vascular insufficiency. These ischaemic lesions are thought to result from occlusion of dermal or subcutaneous arteries, often leading to poor wound healing and an increased risk of secondary infections. The presence of cutaneous findings in GCA should prompt urgent dermatologic evaluation and biopsy to confirm the underlying pathology and guide appropriate management.

## References

[1] Chacko JG, Chacko JA, Salter MW (2015). Review of giant cell arteritis. Saudi J Ophthalmol.

[2] Wagener HP, Hollenhorst RW (1958). The ocular lesions of temporal arteritis. Am J Ophthalmol.

[3] Liu GT, Glaser JS, Schatz NJ, Smith JL (1994). Visual morbidity in giant cell arteritis. Clinical characteristics and prognosis for vision. Ophthalmology.

[4] Choi JH, Shin JH, Jung JH (2019). Arteritic anterior ischemic optic neuropathy associated with giant-cell arteritis in Korean patients: a retrospective single-center analysis and review of the literature. J Clin Neurol.

[5] Hunder GG, Bloch DA, Michel BA (1990). The American College of Rheumatology 1990 criteria for the classification of giant cell arteritis. Arthritis Rheum.

[6] (2024). Giant cell arteritis (temporal arteritis). https://emedicine.medscape.com/article/332483-workup.

[7] Prieto-Peña D, Castañeda S, Atienza-Mateo B, Blanco R, González-Gay MÁ (2021). A review of the dermatological complications of giant cell arteritis. Clin Cosmet Investig Dermatol.

[8] Abukanna AM, Alanazi YF, Alanazi FW (2023). Updates on the prognosis of giant cell arteritis: a systematic review. Cureus.

[9] Bowling K, Rait J, Atkinson J, Srinivas G (2017). Temporal artery biopsy in the diagnosis of giant cell arteritis: does the end justify the means?. Ann Med Surg (Lond).

[10] Hocevar A, Rotar Z, Jese R, Semrl SS, Pizem J, Hawlina M, Tomsic M (2016). Do early diagnosis and glucocorticoid treatment decrease the risk of permanent visual loss and early relapses in giant cell arteritis: a prospective longitudinal study. Medicine (Baltimore).

[11] Mazlumzadeh M, Hunder GG, Easley KA (2006). Treatment of giant cell arteritis using induction therapy with high-dose glucocorticoids: a double-blind, placebo-controlled, randomized prospective clinical trial. Arthritis Rheum.

[12] Spiera RF, Mitnick HJ, Kupersmith M, Richmond M, Spiera H, Peterson MG, Paget SA (2001). A prospective, double-blind, randomized, placebo controlled trial of methotrexate in the treatment of giant cell arteritis (GCA). Clin Exp Rheumatol.

[13] Hachulla E, Boivin V, Pasturel-Michon U (2001). Prognostic factors and long-term evolution in a cohort of 133 patients with giant cell arteritis. Clin Exp Rheumatol.

[14] Uriu SA, Reinecke RD (1973). Temporal arteritis, steroid therapy, and pulmonary emboli. Arch Ophthalmol.

